# Supplementary material for: New perspectives on stress and negative emotions: Positive effects on adolescent learning, memory, and mental health
Source: PLoS One. 2025 Nov 3;20(11):e0336128. doi: 10.1371/journal.pone.0336128 (PMC12582471; doi:10.1371/journal.pone.0336128)
Supplement: Attachment — Submitted filename: Response to Reviewers.docx [file pone.0336128.s002.docx]

**ACADEMIC EDITOR:**

**Scientific:**

Consider increasing the sample size or provide a power analysis to justify the current size.

We sincerely thank the editor for raising this important methodological concern. In response, we have conducted a comprehensive power analysis, based on empirically observed effect sizes (η² = 0.548, Cohen's f = 1.101), which robustly demonstrates that our sample size (N = 53; 17–18 participants per group) is adequate (Figure R1).

Figure. R1 The posterior power analysis in three groups

The statistical evidence indicates high sensitivity, with post-hoc analysis showing >99.9% statistical power (α = 0.05, two-tailed), substantially exceeding conventional thresholds (Figure R2). Additionally, the design maintains over 80% power even under conservative assumptions. We have included the results of the power analysis in the Methods and Results and further discussed the sample size considerations in the Discussion.

Figure. R2 The effect size sample size heatmap shows that the current design (f=0.897, n=17.65) is located in the high-efficiency region (red field)

Include detailed demographic information, such as socioeconomic status and academic performance, to contextualize the findings.

We sincerely appreciate the editor's insightful suggestion to enhance the contextualization of our findings through detailed demographic information. In the revised manuscript, Table 1 now comprehensively presents participant characteristics, including age distribution and academic performance metrics. However, we acknowledge that our original survey instrument did not include questions on socioeconomic status, which is a limitation of the study. Most of our participants were third-year repeat students, which may indicate that their family income is generally at the societal average. We have addressed this limitation in the discussion to provide further context.

Report effect sizes for key statistical comparisons and explore potential non-linear relationships in the data.

We sincerely appreciate the reviewer’s insightful suggestions regarding the reporting of effect sizes and the exploration of potential non-linear relationships in our data. In the revised manuscript, we have meticulously documented all relevant effect sizes, including partial eta squared (η²) for ANOVAs and Cohen's d for t-tests, throughout the Results section. Additionally, we have supplemented the Results section with relevant content to provide further context for these findings.

Furthermore, we conducted a comprehensive analysis of participants' memory sequence scores using a 2 (Time: pre/post) × 3 (Group: Control/Low-Stress/High-Stress) repeated-measures ANOVA, as illustrated in Figure 3A. In addition to these analyses, we examined non-linear relationships between anxiety and memory performance, which revealed a significant inverted U-shaped relationship between changes in anxiety and changes in memory scores, as depicted in Figure 3B. These results underscore the complex interplay between anxiety levels and memory performance. We have included relevant content in the Results.

Strengthen the integration of phenomenological concepts by explicitly linking them to empirical outcomes.

We sincerely appreciate your valuable feedback. In response, we have revised the Discussion section to more clearly articulate the connection between phenomenological concepts and our empirical findings, thereby enhancing the theoretical coherence and interpretative depth of our analysis.

Discuss the limitations of the murine stress model, particularly the differences between physical restraint and human psychological stress.

We sincerely thank you for your helpful and insightful suggestion. In response, we have added a discussion of the limitations associated with the two stress models—subjective stress appraisal in adolescents and physiological stress measurement in mice—in the penultimate paragraph of the Discussion section.This addition helps clarify the scope and interpretative boundaries of our findings across both human and animal models.

**Writing:**

Simplify complex phenomenological passages to improve accessibility for a broader audience.

We sincerely appreciate your valuable feedback. We have revised the phenomenological discussion in the Discussion section based on your valuable suggestion. Specifically, we have simplified the theoretical exposition and strengthened the articulation of its relevance to our experimental findings. These revisions can be found in the revised Discussion section. In addition, we have elaborated on the necessity of employing a phenomenological perspective, which we see as offering an essential first-person complement to empirical research.

Remove redundant sections, such as repeated discussions of high stress effects, to enhance clarity and conciseness.

We sincerely appreciate your comment. Following your suggestion, we have removed the redundant paragraphs to improve the clarity and conciseness of the manuscript.

Ensure all figures (1–6) are included in the final submission and are clearly labeled for reader comprehension.

We thank the editor for this important reminder. We have carefully verified that all six figures (Figures 1–6) are included in the final submission and are clearly labeled to enhance reader comprehension.

Address minor grammatical errors to polish the manuscript.

We appreciate the editor's meticulous attention to manuscript quality. We have conducted detailed grammatical checks throughout the manuscript to ensure language precision.

**Data and Ethics:**

Address data availability restrictions by exploring options for public sharing or providing a stronger justification for limited access.

We would like to thank the editor for highlighting the importance of data accessibility requirements. In compliance with journal policies, we have updated the Data Availability Statement to confirm that all primary datasets are now fully accessible. Specifically: Behavioral metrics and analyzed results are embedded within the manuscript. De-identified metadata are provided in the Supplementary Materials.

This comprehensive approach ensures the protection of participant confidentiality in accordance with ethical protocols while facilitating transparency and reproducibility in our research.

Expand the ethics statement to include specific details on animal welfare (e.g., anesthesia methods) and informed consent processes, particularly for minors.

Thank you to the reviewer for your thorough review and valuable suggestions. We have expanded the Ethics Statement in accordance with your comments, specifically enhancing the descriptions of animal welfare protocols and the informed consent processes for human subjects. All modifications align fully with institutional and international standards. As clearly stated in the Methods section, this study exclusively enrolled adult participants, thereby excluding minors. All ethical considerations have been updated in the manuscript.

**Model Integration:**

Clearly link human and animal findings in the Discussion section, such as connecting corticosterone changes to memory improvement.

We sincerely thank you for your thoughtful suggestion. In response, we have strengthened our discussion of the two stress models—human and animal—as well as their associated mechanisms, with relevant revisions made in both the Introduction and Discussion sections.These additions aim to clarify the rationale and implications of employing both models in our investigation of stress and memory.

Cite relevant studies to support the combined human-animal approach, strengthening the manuscript’s theoretical framework.

Thank you for your valuable suggestion. In the revised Discussion section, we have incorporated relevant studies that combine human and animal models to strengthen the justification for our dual-model approach. Additionally, following your comments—as well as those from other reviewers—we have further analyzed both the internal coherence and the limitations of integrating these two types of models within a single research framework. These revisions aim to provide a more balanced and critically informed perspective on the cross-species applicability of our findings.

**Journal Requirements:**

When submitting your revision, we need you to address these additional requirements.

1.Please ensure that your manuscript meets PLOS ONE's style requirements, including those for file naming. The PLOS ONE style templates can be found at https://journals.plos.org/plosone/s/file?id=wjVg/PLOSOne_formatting_sample_main_body.pdf and <https://journals.plos.org/plosone/s/file?id=ba62/PLOSOne_formatting_sample_title_authors_affiliations.pdf>

We confirm full compliance with PLOS ONE style requirements in the revised submission. All document formatting adheres to the journal templates.

2. Please provide additional details regarding participant consent. In the ethics statement in the Methods and online submission information, please ensure that you have specified (1) whether consent was informed and (2) what type you obtained (for instance, written or verbal, and if verbal, how it was documented and witnessed). If your study included minors, state whether you obtained consent from parents or guardians. If the need for consent was waived by the ethics committee, please include this information.

If you are reporting a retrospective study of medical records or archived samples, please ensure that you have discussed whether all data were fully anonymized before you accessed them and/or whether the IRB or ethics committee waived the requirement for informed consent. If patients provided informed written consent to have data from their medical records used in research, please include this information.

We sincerely appreciate the journal's meticulous attention to ethical documentation. We have supplemented detailed descriptions of informed consent procedures in both the Methods section and Ethics Statement, confirming written consent acquisition from all adult participants with explicit exclusion of minors, consistent with Institutional Review Board requirements.

3. We note that you have indicated that there are restrictions to data sharing for this study. For studies involving human research participant data or other sensitive data, we encourage authors to share de-identified or anonymized data. However, when data cannot be publicly shared for ethical reasons, we allow authors to make their data sets available upon request. For information on unacceptable data access restrictions, please see http://journals.plos.org/plosone/s/data-availability#loc-unacceptable-data-access-restrictions.
Before we proceed with your manuscript, please address the following prompts:
a) If there are ethical or legal restrictions on sharing a de-identified data set, please explain them in detail (e.g., data contain potentially identifying or sensitive patient information, data are owned by a third-party organization, etc.) and who has imposed them (e.g., a Research Ethics Committee or Institutional Review Board, etc.). Please also provide contact information for a data access committee, ethics committee, or other institutional body to which data requests may be sent.

b) If there are no restrictions, please upload the minimal anonymized data set necessary to replicate your study findings to a stable, public repository and provide us with the relevant URLs, DOIs, or accession numbers. Please see http://www.bmj.com/content/340/bmj.c181.long for guidelines on how to de-identify and prepare clinical data for publication. For a list of recommended repositories, please see https://journals.plos.org/plosone/s/recommended-repositories. You also have the option of uploading the data as Supporting Information files, but we would recommend depositing data directly to a data repository if possible.
Please update your Data Availability statement in the submission form accordingly.

We thank the journal for their guidance on data availability. All de-identified datasets have been uploaded as Supporting Information files accompanying this submission, ensuring full reproducibility while maintaining participant confidentiality per ethical restrictions. The Data Availability Statement has been updated accordingly.

4. In the online submission form, you indicated that data from the First Affiliated Hospital of Xi’an Jiaotong University are available from the corresponding author on reasonable request upon aporoval by the hospital.

All PLOS journals now require all data underlying the findings described in their manuscript to be freely available to other researchers, either 1. In a public repository, 2. Within the manuscript itself, or 3. Uploaded as supplementary information.
This policy applies to all data except where public deposition would breach compliance with the protocol approved by your research ethics board. If your data cannot be made publicly available for ethical or legal reasons (e.g., public availability would compromise patient privacy), please explain your reasons on resubmission and your exemption request will be escalated for approval.

We appreciate the policy clarification. The manuscript's supporting datasets have been deposited as supplementary files to ensure public availability, superseding the initial submission form note regarding institutional data sharing protocols. This open-access format satisfies PLOS ONE guidelines while preserving participant anonymity per ethical requirements.

5. [Please remove all personal information, ensure that the data shared are in accordance with participant consent, and re-upload a fully anonymized data set.

Note: spreadsheet columns with personal information must be removed and not hidden as all hidden columns will appear in the published file.

Additional guidance on preparing raw data for publication can be found in our Data Policy (https://journals.plos.org/plosone/s/data-availability#loc-human-research-participant-data-and-other-sensitive-data) and in the following article: http://www.bmj.com/content/340/bmj.c181.long.](http://www.bmj.com/content/340/bmj.c181.long.)

We confirm strict adherence to participant privacy protection by scrutinizing all shared datasets, removing identifiable information (e.g., names) prior to resubmission. The fully anonymized data files have replaced initial uploads, thus ensuring alignment with participant consent protocols and journal guidelines.

[Note: HTML markup is below. Please do not edit.]
Reviewers' comments:
Reviewer's Responses to Questions

**Comments to the Author**
1. Is the manuscript technically sound, and do the data support the conclusions?
The manuscript must describe a technically sound piece of scientific research with data that supports the conclusions. Experiments must have been conducted rigorously, with appropriate controls, replication, and sample sizes. The conclusions must be drawn appropriately based on the data presented.

Reviewer #1: Partly

Reviewer #2: Partly

2. Has the statistical analysis been performed appropriately and rigorously?

Reviewer #1: Yes

Reviewer #2: Yes

3. Have the authors made all data underlying the findings in their manuscript fully available?
The [PLOS Data policy](http://track.editorialmanager.com/CL0/http:%2F%2Fwww.plosone.org%2Fstatic%2Fpolicies.action%23sharing/1/010f01970aa64981-e7cd50a2-8866-4cc7-bfcc-6c1a50e1c89b-000000/G7Oa1eOId5E_fOLCxheaU6-VCkjfEu-Nk1c7-8AI-cw=212) requires authors to make all data underlying the findings described in their manuscript fully available without restriction, with rare exception (please refer to the Data Availability Statement in the manuscript PDF file). The data should be provided as part of the manuscript or its supporting information, or deposited to a public repository. For example, in addition to summary statistics, the data points behind means, medians and variance measures should be available. If there are restrictions on publicly sharing data—e.g. participant privacy or use of data from a third party—those must be specified.

Reviewer #1: No

Reviewer #2: Yes

4. Is the manuscript presented in an intelligible fashion and written in standard English?
PLOS ONE does not copyedit accepted manuscripts, so the language in submitted articles must be clear, correct, and unambiguous. Any typographical or grammatical errors should be corrected at revision, so please note any specific errors here.

Reviewer #1: Yes

Reviewer #2: Yes

Reviewers' comments:
Reviewer's Responses to Questions

**Comments to the Author**

5. Review Comments to the Author
Please use the space provided to explain your answers to the questions above. You may also include additional comments for the author, including concerns about dual publication, research ethics, or publication ethics. (Please upload your review as an attachment if it exceeds 20,000 characters)

Reviewer #1: In this manuscript, the authors investigate the effects of stress on memory performance and stress levels, hypothesizing that moderate stress levels may enhance memory recall - a finding with potential implications for educational settings and other environments. To test their hypothesis, the study combines language-based memory tests in human participants and rodent behavioral assays of emotional and contextual (associative) learning.
For the human cohort, the authors recruited 53 middle school students of both sexes and assessed verbal memory performance under three stress conditions: a control group with no stress induction, a mild stress group where participants were informed of potential proctor observation, and a high stress group tested under direct proctor supervision with an on-screen countdown timer.
In parallel, mouse experiments evaluated associative memory using a passive avoidance test across three groups with stress levels manipulated through differential restraint durations: a high-stress group (HSG) subjected to 12-hour daily restraint for 10 days, a low-stress group (LSG) with 4-hour daily restraint, and a non-restrained control group (NSG). In addition to behavioral readouts, the study measured HPA axis hormone levels to assess physiological stress responses.
Key findings revealed that moderate stress enhanced memory performance compared to controls, while high stress caused significant impairment. Although the manuscript presents these findings clearly, several methodological considerations - detailed in the following review - should be addressed to strengthen the study's validity and impact.

Introduction & Background
1.The claim that "adolescents experience heightened emotional volatility due to hormonal fluctuations and psychosocial stressors" needs a supporting reference.

We thank the reviewer for highlighting the need for supporting literature. We have supplemented the introduction with a key reference:

Revised text (Introduction):

"Adolescents experience heightened emotional volatility due to neuroendocrine reorganization during puberty. As detailed in Smith et al. [8], interactions between stress-responsive hormones (CRH, GnRH, dopamine, serotonin) and psychosocial stressors amplify sensitivity to environmental challenges, resulting in increased emotional lability."

2.Regarding Vogel [9], the statement that "acute stress promotes directed learning in 61 participants" is unclear - why do the authors specify the exact number without context (e.g., total sample size, study design)?

Thank you for your helpful suggestion. In the revised manuscript, we have expanded the description of the Vogel et al. study to clarify key aspects of the experimental design. Specifically, we now report that the study involved 61 participants who were randomly assigned to either a standardized stress condition (the Socially Evaluated Cold Pressor Test) or a non-stressful control. Participants learned stimulus-response associations either through explicit instruction or trial-and-error. The study found that acute stress selectively enhanced performance in the instructed learning condition, which was linked to elevated cortisol levels and autonomic nervous system activation. These details have been added to improve clarity and transparency.

Human Study Methodology
4.With respect to the recruitment of high school students from a middle school is confusing. Please clarify the mean age, standard deviation, and sex distribution as you are only mentioning an age threshold (>18 - do you mean <18?).

We sincerely apologize for any confusion regarding our recruitment process. To clarify, our study exclusively recruited participants from a senior high school, specifically targeting students in their final year, aged 18 to 19 years. In response to your request for detailed demographic information, the mean age of the participants is 18.17 years (± 0.43 SD), with a sex distribution of 39.62% female and 60.38% male. We have explicitly detailed these parameters in Table 1 of the manuscript. Additionally, we have revised the text to address the aspects that may have caused confusion, ensuring that the recruitment criteria and participant demographics are clearly articulated. We appreciate your attention to this detail, as it is essential for understanding the demographic characteristics of our sample.
5.The "Memory Phase" in the pre-test procedure could be named "Memory Encoding Phase" in line with the general vocabulary in the field. The description of word presentation (5 sec/word, 3 sec intervals) needs better formatting for readability.
We would like to express our gratitude to the reviewer for the valuable suggestions. In response to your feedback, we have revised the term "Memory Phase" to "Memory Encoding Phase" to align with the standard terminology in the field. Additionally, we have improved the formatting of the word presentation description for better readability. To further clarify the experimental procedure, we have also included Figure 1, which outlines the steps and flow of the experiment in detail. We believe these modifications enhance the clarity and comprehensibility of the manuscript.
Mouse Model & Behavioral Tests
6.The stress induction protocol (restraint for 4h vs. 12h/day) should be supported by references validating this method for inducing different anxiety levels.

We appreciate the reviewer for highlighting this important methodological consideration. Our review indicates that there is no consensus on the optimal duration for restraint stress, with protocols varying widely from 20 minutes to 24 hours[1-6]. While 6-hour restraints are frequently reported in the literature, we specifically selected 4-hour and 12-hour durations based on their biological relevance and operational feasibility.The 4-hour restraint was chosen because it effectively induces physiological stress markers while maintaining natural feeding rhythms (mice typically consume over 80% of their daily intake during the dark phase). This duration minimizes circadian disruption when administered during the light phase and allows for practical implementation within standard laboratory hours.The 12-hour protocol was selected as it encompasses a complete light-dark cycle, capturing ultradian rhythm disruptions without necessitating overnight staffing. This duration also models prolonged stress exposure scenarios that are relevant to chronic stress in humans.

In the revised manuscript, we have included supporting references in the Methods section to validate our selection of these durations.

*1.	Eom T-M, Kwon H-H, Shin N, Kim D-W, Fang Z, Seol I-C, et al. Traditional Korean herbal formulae, Yuk-Mi-Ji-Hwang-Tang, ameliorates impairment of hippocampal memory ability by chronic restraint stress of mouse model. Journal of Ethnopharmacology. 2020;260:113102. doi: 10.1016/j.jep.2020.113102. PubMed PMID: 32544420; PubMed Central PMCID: PMCQ1.*

*2.	Shanker S, Saroj N, Cordova EJ, Jarillo-Luna RA, López-Sánchez P, Terrón JA. Chronic restraint stress induces serotonin transporter expression in the rat adrenal glands. Mol Cell Endocrinol. 2020;518:110935. doi: 10.1016/j.mce.2020.110935. PubMed PMID: 32659440; PubMed Central PMCID: PMCQ2.*

*3.	Pesarico AP, Carceller H, Guirado R, Coviello S, Nacher J. Long term effects of 24-h-restraint stress on the connectivity and structure of interneurons in the basolateral amygdala. Prog Neuropsychopharmacol Biol Psychiatry. 2022;115:110512. doi: 10.1016/j.pnpbp.2022.110512. PubMed PMID: 35066055; PubMed Central PMCID: PMCQ1.*

*4.	Chiba S, Numakawa T, Ninomiya M, Richards MC, Wakabayashi C, Kunugi H. Chronic restraint stress causes anxiety- and depression-like behaviors, downregulates glucocorticoid receptor expression, and attenuates glutamate release induced by brain-derived neurotrophic factor in the prefrontal cortex. Prog Neuropsychopharmacol Biol Psychiatry. 2012;39(1):112-9. doi: 10.1016/j.pnpbp.2012.05.018. PubMed PMID: 22664354; PubMed Central PMCID: PMCQ1.*

*5.	Luo S, Long H, Lou F, Liu Y, Wang H, Pu J, et al. Chronic restraint stress promotes oral squamous cell carcinoma development by inhibiting ALDH3A1 via stress response hormone. BMC Oral Health. 2024;24(1):43. doi: 10.1186/s12903-023-03787-1. PubMed PMID: 38191346; PubMed Central PMCID: PMCQ1.*

*6.	Molina P, Andero R, Armario A. Restraint or immobilization: A comparison of methodologies for restricting free movement in rodents and their potential impact on physiology and behavior. Neurosci Biobehav Rev. 2023;151:105224. doi: 10.1016/j.neubiorev.2023.105224. PubMed PMID: 37156310; PubMed Central PMCID: PMCQ1.*

7.The claim that "central area exploration (anxiety marker) was higher in HSG" is problematic. Increased center exploration is typically indicating lower anxiety (thigmotaxis, or wall-hugging, reflects anxiety). Your findings may be explained by HSG’s higher locomotion activity - thus, consider alternative interpretations.

Thank you for your thorough review and valuable feedback on our manuscript. We greatly appreciate your insights regarding the interpretation of the elevated central zone exploration observed in the HSG group.

In response to your comments, we have revised the relevant section to clarify our findings, as per your suggestion. While the HSG group exhibited a significantly higher percentage of central exploration (28.97%) compared to the LSG (23.33%) and NSG (23.26%) groups (p < 0.001), we emphasize that this result should be interpreted with caution. We have noted that the HSG group also demonstrated increased locomotor activity (10.62 cm/s vs. 8.11 cm/s). Therefore, we suggest that the heightened central exploration in the HSG group may reflect increased locomotor activity rather than a direct indication of reduced anxiety levels.
Passive Avoidance Test:
8. The term "error" is undefined and an unusual use of language in this context - is this the number of entries into the dark chamber? Labeling this as an "error" is unusual; it could reflect altered risk assessment, associative learning deficits, or differences in shock sensitivity in mice and general differences in memory recall.

We thank the reviewer for this important semantic correction. The term "errors" has been replaced throughout the manuscript and figure with "entries into the dark chamber" to ensure clarity and objectivity. We agree that these entries reflect complex behavioral phenotypes that may involve dysregulated risk assessment, impaired associative learning, altered nociception, and other factors. We have incorporated these multidimensional interpretations into the Discussion.
9. Reporting duration in zones (not just latency/entries) would strengthen the analysis.
We appreciate the reviewer's constructive suggestion regarding the inclusion of duration measurements in each zone. We have now systematically incorporated these duration metrics into our analysis, which provides valuable complementary evidence to the traditional measures of latency and entries. The results are illustrated in the revised Figure 4C.
Conceptual Issues
10. The sudden discussion of Husserl’s phenomenology ("passive/active domains of cognition") seems off-topic and disrupts the paper’s focus. Either integrate it meaningfully or remove it.

We sincerely thank the reviewer for this insightful suggestion. To improve the readability and argumentative clarity of the manuscript, we have removed certain sections that involved technical discussions of Husserlian phenomenological terminology("passive/active domains of cognition”) and retained only those phenomenological concepts that are directly relevant to our research focus.
11. The description of attention’s "horizon structure" is unclear-how does this relate to the study’s hypotheses or results?

We thank the reviewer for this comment. In the revised manuscript, we have streamlined the description of attentional processes in the Discussion, simplify specialized terminology like "horizon structure." The relationship between attentional modulation and our findings is now explicitly described through empirical observations: moderate stress enhanced task-focused attention, while severe stress caused the change of attentional focus. All clarifications regarding this mechanism are provided in the updated Discussion.
12. It is not clear how the results from the mouse models and from the human studies can be compared as they recruit very different skills (verbal recitation versus contextual memory), and the underlying neural substrates are very likely not comparable.

We appreciate the reviewer’s insightful comment regarding the comparison between results from mouse models and human studies. We acknowledge that verbal recitation in humans and contextual memory in mice involve different cognitive skills and likely engage distinct neural substrates. However, there are important similarities in how these memory systems operate.

For instance, participants' word memorization often relies on contextual strategies, where vocabulary learning and recall demonstrate situational dependence akin to episodic memory processes [1,2]. While human verbal memory and rodent contextual memory differ in their manifestations, both rely on shared brain networks—particularly the hippocampal-prefrontal circuitry that is essential for memory formation [3,4].

Notably, in our adolescent study, we observed that moderate stress can enhance memory performance, particularly in vocabulary recall and content retention. Conversely, both our human and mouse studies indicate that severe stress negatively impacts memory performance. This finding aligns with previous research showing that stress can impair learning and memory, particularly in cognitive declarative (or explicit) tasks, such as verbal recall in humans and spatial memory in rodents [5]. This parallel further underscores the utility of mouse models in elucidating human memory processes.

We have expanded our discussion of these connections and their limitations in both the Introduction and Discussion sections. Future research will specifically investigate the mechanistic links between verbal and contextual memory systems. Thank you again for your valuable feedback.

*1.	Vigliocco G, Ponari M, Norbury C. Learning and Processing Abstract Words and Concepts: Insights From Typical and Atypical Development. Top Cogn Sci. 2018;10(3):533-49. doi: 10.1111/tops.12347. PubMed PMID: 29785838; PubMed Central PMCID: PMCQ1.*

*2.	Murphy DH, Hargis MB, Castel AD. Younger and older adults' strategic use of associative memory and metacognitive control when learning foreign vocabulary words of varying importance. Psychol Aging. 2023;38(2):103-16. doi: 10.1037/pag0000730. PubMed PMID: 36757965; PubMed Central PMCID: PMCQ1.*

*3.	Bevandić J, Chareyron LJ, Bachevalier J, Cacucci F, Genzel L, Newcombe NS, et al. Episodic memory development: Bridging animal and human research. Neuron. 2024;112(7):1060-80. doi: 10.1016/j.neuron.2024.01.020. PubMed PMID: 38359826; PubMed Central PMCID: PMCQ1.*

*4.	Allen TA, Morris AM, Mattfeld AT, Stark CEL, Fortin NJ. A Sequence of events model of episodic memory shows parallels in rats and humans. Hippocampus. 2014;24(10):1178-88. doi: 10.1002/hipo.22301. PubMed PMID: 24802767; PubMed Central PMCID: PMCQ3.*

*5.	Kim EJ, Kim JJ. Neurocognitive effects of stress: a metaparadigm perspective. Mol Psychiatry. 2023;28(7):2750-63. doi: 10.1038/s41380-023-01986-4. PubMed PMID: 36759545; PubMed Central PMCID: PMCQ1.*

Reviewer #2: Dear Authors,
This study examines how moderate stress influences adolescent learning and memory through human and murine models, proposing that controlled stress enhances cognitive performance via HPA axis regulation. While innovative in integrating cross-species data, the manuscript requires substantial revisions to meet publication standards. Below are section-specific comments.
Abstract
1.Missing quantitative metrics for key claims (e.g., "significantly better memory" lacks p-values/effect sizes).

Thank you for your thorough review and valuable feedback on our manuscript. We greatly appreciate your comments regarding the missing quantitative metrics. In response to your suggestion, we have now included the relevant p-values and effect sizes for all key claims in the revised manuscript. We believe these additions will provide clearer insights into the significance of our findings.
2.Theoretical implications of "multidimensional emotion assessment" remain vague.
Introduction

We sincerely thank the reviewer for this thoughtful and constructive suggestion. Our study primarily evaluates adolescents’ emotional states from two perspectives: physiological indicators (such as HRV values) and psychological measures (self-report questionnaires). However, we acknowledge that these two aspects may not fully capture the multidimensionality implied by the term. In light of the reviewer’s comment, we have removed the term “multidimensional” from the manuscript to avoid ambiguity and potential overstatement. The revision can be found in the Abstract section of the manuscript.
3.Seminal works on stress theory (e.g., Lazarus & Folkman) are omitted despite critiquing "long-standing views."

We sincerely thank the reviewer for highlighting this important reference. In response, we have added a discussion and citation of Lazarus and Folkman’s seminal work at the end of the first paragraph in the Introduction section. This addition helps to contextualize the cross-disciplinary conceptualizations of stress and strengthens the theoretical foundation of our study.
4.The rationale for focusing solely on acute stress (vs. chronic stress) is unexplained.

We thank the reviewer for raising this important consideration. Our focus on acute stress was driven by several key factors that align with our research objectives and the context of adolescent mental health.

First and foremost, this choice relates to the nature of our experimental design. Specifically, the tasks involving word memorization and recitation are considered acute stressors. Moreover, our selection of task-related stressors aligns with the types of stress most commonly encountered by adolescents in academic settings.Second, acute stress allows for greater mechanistic clarity. By focusing on acute stress, we can precisely isolate the dynamics of the hypothalamic-pituitary-adrenal (HPA) axis without the confounding effects of chronic stress adaptations, such as dendritic atrophy and receptor downregulation. Our murine model demonstrates that even with repeated restraint over a period of 10 days, the acute glucocorticoid response patterns were maintained (see Fig. 6A-B), enabling clear dose-response analysis.Lastly, the relevance of our findings to intervention strategies is a crucial consideration. Acute stress management techniques, such as pre-exam mindfulness practices, offer immediate practical applications for educators and students. In contrast, interventions for chronic stress often require longer-term systemic changes that extend beyond the scope of our study.

Methods
Human Participants:
5. No demographic data (gender, socioeconomic status) or screening for pre-existing mental health conditions.

We sincerely appreciate the reviewer’s insightful suggestion to enhance the contextualization of our findings through detailed demographic information. In the revised manuscript, we have included a new Table 1 that comprehensively presents participant characteristics, including age distribution and academic performance metrics.

However, we acknowledge that our original survey instrument did not include questions regarding socioeconomic status and pre-existing mental health conditions, which are important factors that could influence our results. This omission represents a limitation of our study. We have addressed this limitation in the discussion section to provide further context and underscore the potential impact of these factors on our findings.

6. Ethical concerns: Unclear protocol for stress induction in minors despite IRB approval.

We sincerely thank the reviewer for raising important ethical considerations. We would like to clarify that all participants in our study were legal adults, aged 18 to 19 years, and were recruited as high school repeat students. Age documentation was verified and submitted to the ethics committee as part of the review process.

To enhance the transparency of our stress induction protocol, we have made two significant clarifications in the revised manuscript. First, in the Methods section, we explicitly state that stress induction was limited to simulated academic evaluation scenarios, specifically timed proctored tests with performance feedback. These scenarios were intentionally designed to be milder than actual national college entrance exams. Importantly, participants retained the right to terminate their involvement at any time without penalty, and we implemented real-time anxiety monitoring along with on-site psychological support to ensure their well-being throughout the study.

Additionally, we have included Figure 1 in the revised manuscript to provide a visual representation of the entire testing process, thereby enhancing the clarity and intuitiveness of our stress induction protocol.

These revisions ensure that our study fully aligns with ethical standards for research involving adults, and we appreciate the reviewer’s attention to this critical aspect.

Murine Model:
7.Stress induction duration/frequency insufficiently detailed (e.g., intervals between stressors).

We sincerely thank the reviewer for highlighting these important methodological details regarding the stress induction duration and frequency. In response, we have comprehensively revised the Methods section to provide a more detailed account of our stress induction protocols. This includes specific information about the duration of each stressor and the intervals between them.

Furthermore, we have created a new Figure 1, which visually outlines all experimental timelines and procedures. This figure is designed to enhance the reader's understanding of the stress induction process and ensures that all aspects of our methodology are transparent and clearly communicated

8.No justification for excluding female mice from the analysis.

We sincerely appreciate the reviewer’s attention to the inclusion of sex in our study.The decision to exclude female mice was based on established biological complexities in HPA-axis regulation. As demonstrated in prior research [1,2], female rodents exhibit amplified HPA responses due to estradiol-mediated potentiation of glucocorticoid release. These effects are further compounded by cyclic fluctuations of gonadal steroids across the estrous cycle, which introduce significant variance in stress hormone levels independent of experimental manipulations.

Given our focus on precise HPA-axis biomarker quantification (corticosterone/ACTH), the inherent hormonal variability in females would have confounded stress-intensity comparisons between groups. We therefore limited initial analyses to male mice to establish baseline mechanisms without sex-specific interactions. We have explicitly acknowledged this limitation in the Discussion, emphasizing the need for future sex-stratified investigations.

*[1] Oyola MG, Handa RJ. Hypothalamic-pituitary-adrenal and hypothalamic-pituitary-gonadal axes: sex differences in regulation of stress responsivity. Stress. 2017;20(5):476-494. doi:10.1080/10253890.2017.1369523*

*[2] Kokras N, Krokida S, Varoudaki TZ, Dalla C. Do corticosterone levels predict female depressive-like behavior in rodents? J Neurosci Res. 2021;99(1):324-331. doi:10.1002/jnr.24686*

Statistical Analysis:

9. ANOVA results lack post-hoc comparisons (e.g., Tukey’s test) and effect size reporting (η²/Cohen’s d).

We appreciate the reviewer’s insightful feedback regarding the need for post-hoc comparisons and effect size reporting in our ANOVA results. In response to this concern, we have conducted post-hoc Dunnett's tests to further analyze the differences between groups. We have also revised the Results section of the manuscript to include these findings comprehensively. Additionally, we have reported effect sizes using both η² and Cohen’s d, providing a clearer understanding of the magnitude of the observed effects. We believe these enhancements strengthen our analysis and improve the overall quality of the manuscript.

Results

10. Human memory performance data are presented as group averages without individual variability measures (e.g., SD/SE).

We appreciate the reviewer’s commitment to enhancing the clarity of our data presentation. In response, we have supplemented the manuscript with individual variability measures for the human memory performance data. The results are now presented as mean ± SE, and we have conducted a thorough review to ensure that these measures are consistently reported throughout the manuscript.

11. Murine HPA axis hormone levels (corticosterone, ACTH) are not directly correlated with human outcomes.

We appreciate the reviewer's important observation regarding cross-species HPA-axis comparisons. While murine corticosterone levels cannot be directly equated to human cortisol dynamics, our study aligns with established evidence demonstrating conserved stress-memory mechanisms across species. Previous research confirms that stress similarly modulates hippocampus-dependent memory in humans and rodents through glucocorticoid signaling[1, 2], and our murine corticosterone responses correspond to behavioral outcomes consistent with these fundamental principles.

Ethical constraints precluded HPA-axis monitoring in our adolescent cohort. We have strengthened the discussion of cross-species memory mechanisms in the revised manuscript, explicitly noting in the Limitations section that direct hormonal comparisons remain constrained by species-specific physiological differences.

*1.	Kim EJ, Kim JJ. Neurocognitive effects of stress: a metaparadigm perspective. Mol Psychiatry. 2023;28(7):2750-63. doi: 10.1038/s41380-023-01986-4. PubMed PMID: 36759545; PubMed Central PMCID: PMCQ1.*

*2.	Chiba S, Numakawa T, Ninomiya M, Richards MC, Wakabayashi C, Kunugi H. Chronic restraint stress causes anxiety- and depression-like behaviors, downregulates glucocorticoid receptor expression, and attenuates glutamate release induced by brain-derived neurotrophic factor in the prefrontal cortex. Prog Neuropsychopharmacol Biol Psychiatry. 2012;39(1):112-9. doi: 10.1016/j.pnpbp.2012.05.018. PubMed PMID: 22664354; PubMed Central PMCID: PMCQ1.*

Discussion

12. Discrepancies between human and murine results (e.g., HPA regulation mechanisms) are unaddressed.

We thank the reviewer for highlighting this important consideration. We have substantially strengthened the discussion of cross-species connections throughout the manuscript to address potential discrepancies.

In the revised Introduction, we articulate how both human verbal recall and rodent contextual memory engage conserved hippocampal-prefrontal networks critical for memory formation . The Discussion further establishes that although task modalities differ, both paradigms demonstrate parallel stress effects on memory – moderate stress enhances performance while severe stress causes impairment through shared neural mechanisms.

Critically, we explicitly state in the Limitations that murine HPA-axis measures cannot be directly extrapolated to human outcomes:

"Additionally, while murine corticosterone levels provide valuable mechanistic insights, they should not be directly equated with human cortisol dynamics due to species-specific physiological differences."

These revisions clarify that our comparative approach focuses on functional behavioral outcomes (memory enhancement/impairment patterns) and conserved neurocircuitry, not direct hormonal correlations.

13. Over interpretation of "38% improvement in learning efficiency" without mechanistic evidence.

We thank the reviewer for their comment regarding the "38% improvement in learning efficiency." We acknowledge that we may not have explicitly highlighted this data in the manuscript. To enhance readability and clarity, we have reviewed the manuscript to eliminate any similar descriptions that lack supporting evidence. We appreciate the reviewer’s feedback, which has helped us improve the rigor of our presentation.

Conclusion

14. Educational policy recommendations are premature given the small human sample (n=53) and single-institution design.

We appreciate the reviewer's valid concern regarding the premature nature of educational policy recommendations based on our single-center adolescent cohort (n=53). We fully acknowledge this limitation and have removed all educational policy implications from the Discussion and Conclusion sections. The revised manuscript now reframes the implications to focus exclusively on mechanistic insights into stress-memory interactions. Additionally, we explicitly state in the Discussion that broader educational applications necessitate multi-center replications with socioeconomically diverse samples.
